# Evaluation of a Participatory Action Project to Address Opioid Misuse: Breaking Down Barriers Through Partnership Processes

**DOI:** 10.5130/ijcre.v17i1.9202

**Published:** 2024-12-19

**Authors:** Carlin Rafie, Emily Zimmerman, Dawn Reed, Angelina Hargrove

**Affiliations:** 1 Department of Human Nutrition, Foods and Exercise, Virginia Polytechnic Institute and State University; 2 Department of Epidemiology, School of Public Health, Virginia Commonwealth University; 3 UNC Health, North Carolina; 4 Center for Public Health Practice and Research, Virginia Polytechnic Institute and State University

**Keywords:** Community Engaged Research, Community Based Participatory Research, Partnership Synergy, Opioid Epidemic, Community Based Participatory Research Model, Ripple Effects Mapping

## Abstract

Community based participatory research and participatory action research are increasingly being used to engage communities in addressing social and health disparities. There is a need to develop broadly applicable evaluation methods that can be used across participatory project environments to identify the processes critical for addressing complex public health issues, as well as the productiveness of community research partnerships. We present a case study of a community participatory project conducted over three years and our evaluation approach. We used the Community Based Participatory Research Conceptual Model as the framework for the evaluation surveys (n=9) and interviews (n=7) with project participants, querying perspectives on the four model domains: community context, partnership processes, intervention and research and outcomes. In addition, we conducted a Ripple Effects Mapping (REM) exercise with ten community members to determine the broader impacts of the project on the community. This mixed-methods approach permitted us to confirm findings from quantitative surveys with qualitative findings from interviews and the REM. Key processes identified as facilitators to a productive partnership and positive outcomes include a context of trust, effective implementation of processes that establish equitable partner relationships and partnership synergy, a clearly defined focus for the partnership and a structured participatory research method that helped break down silos and mobilise the community for action. Our project evaluation approach, combining the CBPR model and REM, guided measurement of common metrics that are key to effective community engagement as well as exploration of unanticipated outcomes.

## Introduction

Community based participatory research (CBPR) and participatory action research (PAR) are now widely accepted as legitimate methods for addressing social and health inequalities in marginalised communities (McElfish et al. 2015; Wallerstein & Duran 2006). In these approaches, researchers and community representatives work together to build on the strengths of the community, sharing responsibility for the research process. CBPR and PAR have a different epistemological foundation than other research methods (Meyer 2000). They seek to integrate action, theory, practice and critical self-reflection in the pursuit of pragmatic solutions to community concerns (Jagosh et al. 2012; Reason & Bradbury 2001). As a way to address public health concerns, the collaboration between academics and community members focuses on enhancing understanding of a health concern, as well as implementing tailored interventions and policy changes based on what has been learned (Israel et al. 2010).

Despite the increased use of participatory research, there are fewer available standardised evaluation measures and methods for CBPR and PAR compared to conventional research approaches. Several recent reviews have identified measures to evaluate the quality and outcomes of community engaged research collaborations (Luger, Hamilton & True 2020; Tigges et al. 2019). With the exception of a few cases, such as the work of Oetzel et al. (2015), the majority of measures have not been thoroughly examined or tested (Israel et al. 2020; Sandoval et al. 2012). The challenge in evaluating participatory research stems from identifying which aspects of engagement are critical for achieving the desired outcomes (Oetzel et al. 2018). Furthermore, it can be difficult to compare participatory research interventions because evaluation approaches tend to be tailored to the specific context (Meyer 2000). Without validated tools and models, it is difficult to apply evaluation methods across different contexts.

Evaluating the productiveness and structural arrangements of research partnerships is another challenge (Belone et al. 2016). Multidisciplinary processes are difficult to manage and their outcomes are often unclear or subject to interpretation. Important process indicators, such as relationship quality, trust, participation and relational dynamics, can be hard to measure objectively (Höppner 2009; Pohl & Hadorn 2008), yet they are critical because they drive the productivity of the partnership and are important outcomes in their own right. Participatory research practitioners must also reflect on the challenges faced when conducting and evaluating research to improve their understanding of best practices (Dedding et al. 2021) and disentangle the multiple ways that engagement contributes to achieving project aims. Case studies can enhance the knowledge base for participatory research evaluation approaches by illustrating the use of specific frameworks, methods and constructs (Hicks et al. 2012; Reese et al. 2019; Sandoval et al. 2012).

This paper seeks to advance the scientific understanding of participatory research partnerships by highlighting our evaluation results, following a description of the project and its outcomes. We demonstrate the complexities of evaluating participatory research by presenting a case study focusing on the evaluation methods and framework that were employed.

## Background

Opioid misuse is a significant health issue globally. In response to the dramatic increase in opioid dependence and overdose deaths in the United States, the U.S. Department of Health and Human Services (2017) declared the opioid crisis a public health emergency on 26 October 2017. There is substantial variation in community rates of opioid misuse and overdose, and the availability of prevention and treatment services, necessitating community designed solutions in addition to national action. We report on the evaluation of a participatory action project in a rural Virginia community that wanted to further its efforts to tackle the opioid crisis.

Virginia has been greatly impacted by opioid misuse and overdose. The rural community that is the focus of this project, Martinsville City/Henry County, had one of the highest average per capita opioid prescription rates in the U.S. between 2006 and 2012 (U.S. News & World Reports 2017). As reported previously, the community’s opioid overdose death rates were three times higher than the state average in 2016, and the area had the highest rate of opioid overdose emergency room visits statewide in 2018 (Virginia Department of Health n.d.; Zimmerman et al. 2020b). Much like other rural communities, this county had lower levels of educational attainment, higher levels of disability and lower median household incomes (United States Census Bureau 2020). Once a prosperous farming and manufacturing center, the community experienced economic downturn as factories closed through the 1980s, 90s and 2000s.

Despite the significant challenges facing this resilient community, it mobilised to form an opioid task force in 2016. The task force was composed of key community organizations including the police department, behavioral health providers, peer and faith-based recovery programs, the local hospital and representatives from the judicial system. To formalise a process to take action, the task force aligned with an existing community-academic research partnership, Engaging Martinsville (EM). EM decided to use a community-engaged approach, the Stakeholder Engagement in Research Question Development and Prioritization Method (SEED Method), that they had previously used in the community to address a disparity in lung cancer mortality (Rafie et al. 2019). With support from the opioid task force, EM implemented a project using the SEED Method to develop community action plans to address the opioid problem (Zimmerman et al. 2020b).

Involving residents and local stakeholders in action planning is important to ensure that a community’s strategies are based on an understanding of the local situation and reflect the community’s concerns and needs (Kapiriri & Norheim 2002; Lomas et al. 2003). Without community buy-in, many potentially effective strategies, such as harm reduction, may not be achievable (Childs et al. 2021). Currently, there is a mix of top-level leadership approaches, coalition approaches and evidence-based approaches being offered as options for engaging communities. While coalition approaches generally involve a variety of community stakeholders, they may not be founded on participatory values (such as inclusion of people with lived experience) and are not easily transferable to other communities (Windsor 2013).

The SEED Method was designed in response to a need for systematic, participatory engagement methods that engage people with lived experience to create research agendas or action plans. The SEED Method addresses the limitations of effective community stakeholder engagement, including the need for technical training of stakeholders and community capacity building (Hoffman et al. 2010; O’Haire et al. 2011; Oliver et al. 2004), by incorporating research training and providing facilitation tools that lead the teams through the process of stakeholder selection, conceptual modeling and strategy development and prioritization. Participants in past SEED projects indicated that SEED prepared them well for the tasks they were asked to perform and that they had a sense of satisfaction at gaining new skills in the process (Rafie et al. 2019; Zimmerman et al. 2017). Results from previous SEED Method demonstration projects strengthen the evidence that engaging diverse groups of stakeholders in research results in pinpointing more comprehensive priorities for health research, and that SEED has application in identifying and prioritizing actions to address a variety of community health challenges. We anticipated that applying the SEED Method as a participatory action planning tool within this community would result in collaborative actions on community-derived strategies and help strengthen partnerships.

**Figure 1.**
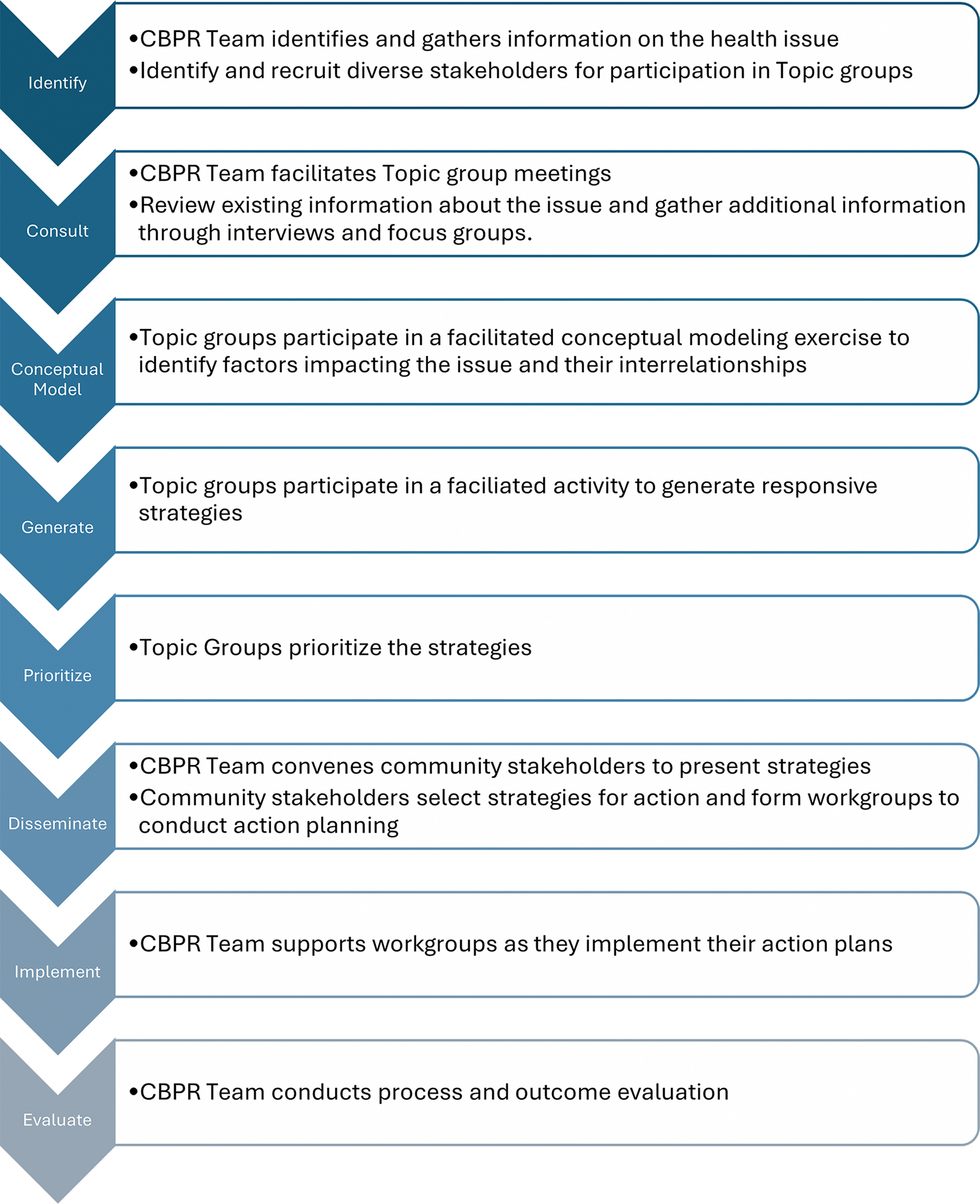
Steps of the SEED Method

The SEED Method combines collaborative, participatory and consultative roles to engage community members in identifying and prioritizing strategies. The method engages stakeholders at three levels: (1) Community Research Team (CBPR Team) – a multidisciplinary partnership that collaboratively leads the project; (2) Topic Groups (TGs) – participatory stakeholders recruited based on their experience and knowledge of the health topic; and (3) SCAN participants – consulting stakeholders who participate in focus groups and interviews. At each level, stakeholders make unique contributions to the project but work together iteratively (Zimmerman et al. 2020a). We modified the SEED Method, originally developed to focus on research question development, to include facilitated steps for action planning and intervention development. Figure 1 outlines steps of the modified SEED Method.

### PARTICIPANT INVOLVEMENT

The project involved a total of 85 community stakeholders at various levels of engagement. Eight community members formed part of the initial participatory community research team, EM. Over the course of the three-year project, five EM team members left due to other obligations and family health issues. At the same time, three new members joined the team to replace those who had left. EM team members included three peer substance misuse counselors, one nurse, two prevention specialists, two medical administrators, two university faculty members and a graduate student. The EM team met weekly at the beginning of the project, decreasing to fortnightly at the end of the first year. At the beginning of the project, most meetings were held in person at a local community facility. In response to the COVID-19 pandemic, team meetings shifted to being fully virtual. The shift in meeting format did not negatively impact the team’s work; in fact, it allowed for more consistent attendance. The community members of the research team were paid an hourly minimum wage for all project-related work.

The EM team chose three stakeholder groups to form the Topic Groups. The team recruited 21 community members for TGs composed of: (1) family and friends impacted by opioid use disorder; (2) service providers (e.g., police, EMT’s); and (3) healthcare providers. TG members were recruited through newspaper advertisements and fliers posted and distributed through email to key organizations. Compared to the community demographics, EM and TG members were slightly overrepresented by individuals of White race with a higher level of education. Their ages ranged between 21 and 64 years, 65 per cent reported having a college degree and 14 per cent identified their race as Black. Each TG met seven times and meetings were facilitated by members of the EM team. Meetings took place at a local healthcare facility. Participants received a stipend ($250) for their participation and food was provided during meetings.

In addition to research teams and TGs, community members were invited to participate in focus groups. Twenty-four community stakeholders participated in four focus groups and provided additional community perspectives on the opioid issue. The TGs and EM team each chose unique stakeholder groups from whom they wanted to obtain information to inform their work. Participants were recruited by the EM team through multimedia advertising. EM conducted the focus groups and summarised the information for presentation to the TGs. Focus group participants received a stipend ($25).

Following the creation and prioritization of strategies by the TGs, they were presented at two community meetings attended by 41 community members. During the meetings, community stakeholders voted on a short list of strategies. The three-hour meetings took place approximately four weeks apart at a local community facility and dinner was provided. The attendees chose four strategies to work on over the next year and formed a work group for each strategy.

A total of 29 stakeholders joined the four working groups that were formed at these community meetings. Some of the stakeholders had participated previously (e.g., as TG members) and some were new to the project. The working groups created their own meeting schedules, but generally met monthly. The working groups met for 12 to 24 months and received continuing support from and communication with the EM team. Initially, most meetings occurred in person, but later transitioned to virtual or other formats in response to the COVID-19 pandemic. Despite this change of meeting format, the teams continued to work effectively together and accomplished their objectives. The EM team arranged quarterly meetings with the work group coordinators to discuss progress and challenges. Bi-annually, EM hosted meetings bringing together all four work groups to update each other on progress and share a meal. Work group members were not compensated for their work on the strategies.

## Evaluation Approach and Methods

We used the CBPR Conceptual Model (UNM College of Population Health n.d.) as the framework to evaluate the effect of the SEED Method on partnership processes and their impact on partnership synergy and project outcomes. This model was developed by Wallerstein and colleagues through a two-year pilot study that looked at how the CBPR process influences or predicts outcomes (Wallerstein et al. 2008; Wallerstein & Duran 2010). The model addresses four CBPR domains (contexts, partnership processes, intervention and research and outcomes) and outlines the potential relationships between each. The first domain consists of contextual factors that shape the nature of the research and the partnership, such as social, structural and political factors. The second domain, partnership processes, includes the partnership structure, members and member relationships, which interact with contextual factors to shape both the intervention and its research design. If partnering practices are effective, they will result in mutual learning, an ability to work together effectively and partner synergy, which impacts outcomes. The third domain consists of the intervention and research processes aligned with CBPR principles. These factors, taken together, result in intermediate system and capacity changes, and ultimately health outcomes (Oetzel et al. 2018). The evaluation tools associated with the CBPR model were validated initially by Oetzel et al. (2015). More recently, Boursaw et al. (2021) evaluated the psychometric properties of seven revised scales that corresponded to the four CBPR model domains. We modified the survey and interview guides developed for the purpose of evaluating constructs within each of these domains (Engage for Equity n.d.; UNM College of Population Health n.d.). The project was approved by the Virginia Tech Institutional Review Board (VT IRB 18–860) and all individuals participating in research procedures provided signed informed consent.

### EVALUATION QUESTIONS AND ANALYSIS

The community research team selected constructs within each of the CPBR model domains that they felt were most relevant to their partnership and selected questions from the survey that focused on these constructs. Within the context domain, we evaluated the social and structural context of our community and our partnership capacity. In the process domain, we focused on partnership structure including the ability to bridge differences, the partnership core values and alignment with community engagement principles. We also included constructs related to relationships, including quality of dialogue, participatory decision making, leadership, resource use and trust. We were particularly interested in evaluating community involvement in research in the intervention and research domain, which has been shown to impact outcomes. Within the outcome domain, we focused on community organization outcomes and partner challenges, health outcomes and project sustainability. In addition, we included two questions about the overall quality of the partnership work and satisfaction with the partnering experience. The final survey consisted of 64 questions that focused on 15 constructs within the four CBPR domains (See Appendix 1). All survey questions were on a Likert scale of either six or seven points, which varied depending on the construct. We calculated the mean score for each construct in a domain. Mean scores of four were considered indicative of a positive response for six-point Likert scales and a mean score of five for seven-point Likert scale constructs. We used the scores to evaluate the effectiveness of the partnership and to inform conversations with the community research team about partnership sustainability and future activities.

The survey was developed for online access using Qualtrics. Project collaborators (members of the research team, TGs and community work groups) were invited to complete the survey through an email invitation approximately 28 months after the start of the project, which had a duration of 36 months. Nine TG members participated in the survey.

### QUALITATIVE INTERVIEWS AND ANALYSIS

Current and former members of the research team were invited to participate in qualitative, one-on-one interviews using a modification of the CBPR Conceptual Model interview guide (Engage for Equity n.d.; See Appendix 2). Seven team members participated in the interviews.

A research assistant who was not associated with the project conducted the interviews. The semi-structured interviews lasted approximately 30 minutes and took place on Zoom. The interview guide queried constructs within each of the four CBPR domains to mirror those included in the survey, and solicited recommendations for improved partnership management and dissemination of project findings. Interviews were recorded and automatically transcribed by Otter.ai, a third-party vendor. Transcriptions were checked for accuracy prior to coding. All coding was conducted using Taguette (version 0.10.1), a third-party vendor, and computer-assisted qualitative data analysis software. We used the framework method for transcript analysis (Gale et al. 2013). Initial coding was conducted by the research assistant under the supervision of a member of the research team. Transcript analysis began with a focus on trust within the partnership, facilitators and barriers to trust, and the impact of trust on the effectiveness of the SEED Method in community action planning.

The research team expanded on the preliminary analysis using the CBPR model domains and constructs as a framework for coding and theme development. An initial code list was created deductively for each domain of the CBPR model, incorporating the codes related to trust developed from the initial analysis. Upon review of each transcript, additional codes were added inductively as appropriate. The finalised codes were grouped by domain and constructs within each domain. They were then categorised into broader themes under each construct. The recommendations made in response to the final interview question, ‘If another group were going to start this kind of partnership, what would you tell them in order to help them be successful?’, were grouped and evaluated for common themes.

### ADDITIONAL EVALUATION METRICS

Several additional assessment tools were used to evaluate the process and outcomes of the study. Demographic information was collected from project collaborators. In addition, EM and TG members were asked to complete a group readiness questionnaire used in previous SEED projects early in their work together. Other data useful for the evaluation included community perspectives on the opioid crisis gathered through focus groups (Hargrove et al. 2022), the strategies developed and prioritised by the TGs and selected for action by community stakeholders, and work group accomplishments, the details of which will be reported elsewhere.

We also conducted a Ripple Effects Mapping exercise with project partners to capture information about both intended and unintended consequences of the project (Chazdon et al. 2017; Emery et al. 2015). We followed the process described by Chazdon et al. (2017). The REM process visually maps intended and unintended impacts of a project and employs four key elements: (1) appreciative inquiry; (2) a participatory approach; (3) interactive group interviewing and reflection; and (4) mind mapping that is used to chart the chain of effects of the project.

Appreciative inquiry is a process of asking unconditional, positive questions that strengthen a system’s positive potential. In the context of REM, appreciative inquiry is used to facilitate the discovery of participants’ best experiences with the program or project, and the unanticipated effects of the project in the community (ripple effects). We employed a participatory approach to the conduct of the REM, involving the research team in the choice of questions asked during the REM, the conduct of the exercise and analysis of the outcomes.

The REM exercise was conducted over a two-hour Zoom meeting. The exercise was facilitated by a member of the research team, assisted by members of EM. We used the online mapping program, Mind Meister [2023], to illustrate the participants’ best experiences and community impacts as they were discussed. We invited diverse participants, including members of the research team, TGs and work groups, as well as focus group and community meeting participants. Ten community members participated in the session.

The exercise began with a brief overview of the REM process, followed by a 15-minute period in which participants interviewed each other. They were provided with specific instructions on how to conduct the interviews. They selected three questions from a longer list of questions developed by the research team. Participants were paired up and directed to Zoom breakout rooms for this activity. After the interview session, participants shared and discussed the most salient points from their interviews. The facilitator used the Mind Meister program to lead the attendees in a process of visualizing the project impacts and illustrating cause-and-effect relationships between them (mind mapping). Using a rippling and theming approach, topics were displayed in the map and similar topics were positioned together under common themes. Participants commented on what they thought came about and any attributions to the causes. A final 15-minute group meeting allowed participants to reflect on how the mapping process made them feel. They also reflected on what was interesting about the map and the story it told.

Following the REM session, minutes from the meeting were reviewed, and information from the map was downloaded in outline format and edited for clarity. Two members of the research team reviewed the notes from the meeting and the map contents and identified themes and REM outcome areas that were informed by the REM guiding questions. Themes and information from the REM exercise were then organised according to the level of impact (i.e., individual, interpersonal/community, environmental).

## Results

**Figure 2.**
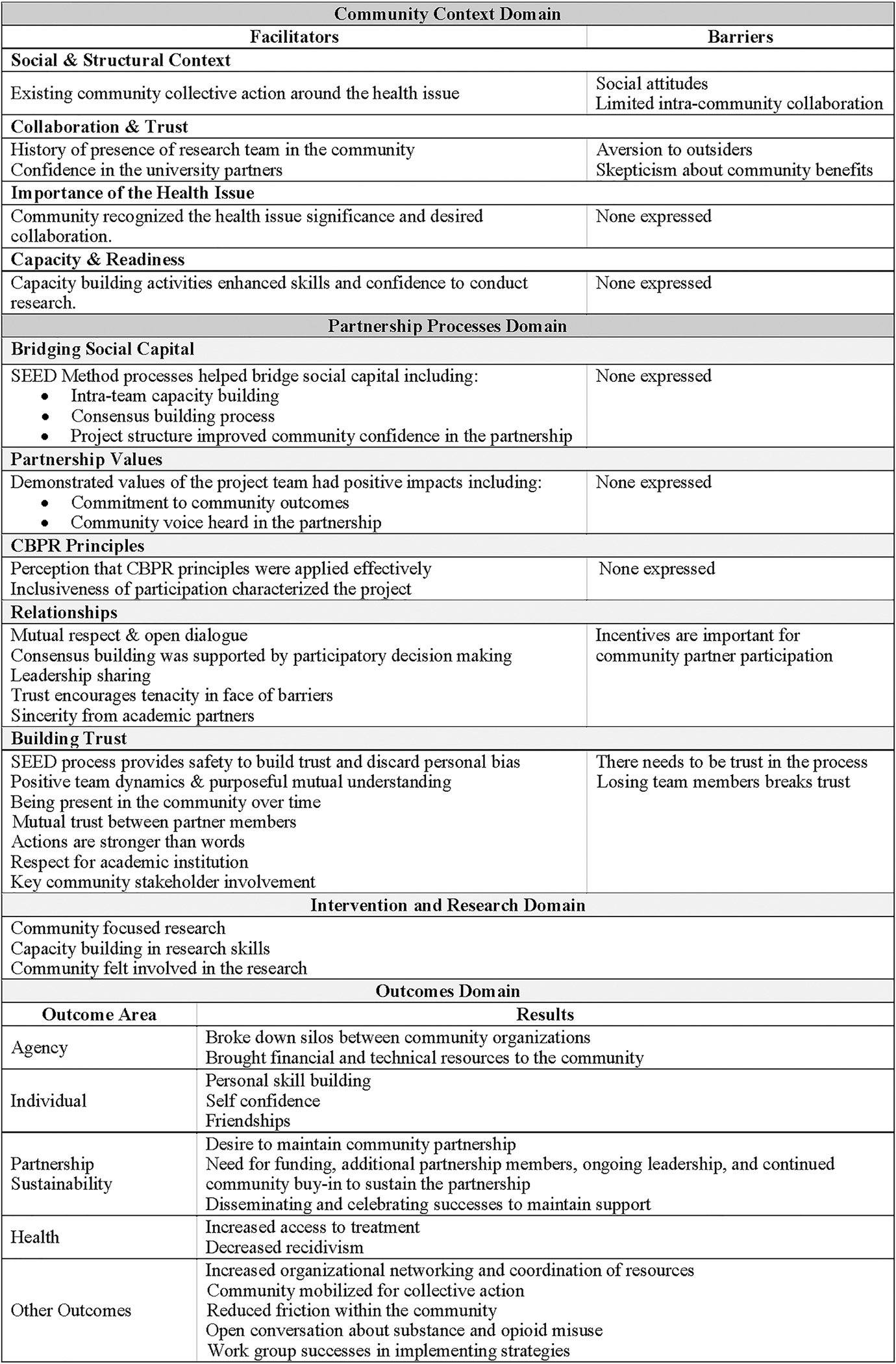
Themes from interviews with the Research Team (N=7) by CBPR Conceptual Model Domain

Key themes from the survey (Tables 1, 2, and 3) and interviews (Figure 2) included the importance of building relationships and trust to the community partnership and partnership synergy, as well as the impact of the structured method of engagement (the SEED Method).

### BUILDING RELATIONSHIPS

Processes for quality and equitable relationships among partners are important for sustainable, productive community partnerships. Factors that promote good partner relationships include quality dialogue, participatory decision making and strong leadership. Survey results show a strong mean score for quality dialogue (5.54/7, s.d. 1.21) among the project partners, which was composed of questions related to mutual respect, good listening and conflict resolution. The project also had strong, effective leadership as reflected in the mean score for this construct (5.1/6, s.d. 0.37), which is associated with team synergy (Table 1).

In interview responses, relationship building is seen as an outcome of treating each other with mutual respect and engaging in participatory decision making.

For instance, when we began our team, we started out by first of all getting to know each other, trying to create an environment where we got to know each other on an equal basis.

But we tried to create an environment where we’re all equal partners in this and that decisions are made equally.

We use first names, we share decision making, and we consider everybody around the table to be an expert in whatever it is that they kind of represent. So, if it’s a community member, they may be an expert.

And once everybody kind of made their argument, a lot of times at that point we could come to an agreement.

So, it’s kind of a combination of getting to know the individuals as persons, but also working together to identify what’s going on in the community, what the needs are and what the interest is in addressing those.

Strong leadership, community inclusion throughout the project, and community members serving as leaders and decision-makers strengthened the partnership.

I don’t have the degrees that other people have, the educational degrees. I’m still made a part of the group. I think that the partners still trust my suggestions and judgments and have made me a part of the whole process.

I think [NAME 1] and [NAME 2] have exemplary leadership, when it comes to some of these situations where like say going into a community that may have some resistance.

I feel that my voice is heard in the meetings. I’ve even helped bring in some other people that I would think that had a lot to offer to this issue, and they are very much welcomed into the group and their voices being heard.

### TRUST

Trust is integral to collaborative projects and provides a foundation for project sustainability and successful outcomes. The survey findings indicate a significant level of trust between those working on the project (mean = 6.29/7, s.d. 0.19) (Table 2). This is significant, as trust and team synergy are associated with project sustainability and outcomes. These positive partnership process scores may be a consequence of the partnership structure that incorporated community engagement principles resulting in strong relationships built on trust.

The importance of trust to the partnership was a repeated theme in the interviews, as well. This included trust between individuals, academic and community partners, and trust in the process. Familiarity, purposeful mutual understanding, being present over time, and ‘doing what you say’ were factors that built trust. Other factors considered important were having a consistent presence in the community throughout the project, especially during challenging times, and the commitment of the partners to each other and to the goals of the project.

So, they, they had to trust us. And yet, we also had to trust them. Because it’s not going to work if it’s just people from the university trying to make something happen. And so, we have to trust them also, that they’re going to put in their part.

So, it’s kind of like this mutual trust, kind of expectations of one another. And then we’re going to see whether or not those actually get fulfilled.

I think that we built a level of at least trust and support for one another, they can come to us, and we can come to them.

And as long as our team continues to be safe, do what we say, right, and be consistent in our presence, we’re going to do what’s on the part of everybody, right?

One thing is you have to trust the structure itself. Like, if you don’t think it’s a good process, then the structure probably isn’t going to help.

Conversely, turnover of team members that can occur with lengthy projects was identified as a hindrance to trust.

But sometimes people just stop participating. And you don’t know why. And I think that kind of challenges trust, even throughout the team.

The history of previous work of the Engaging Martinsville team, and the reputation of the two universities involved in the project, were credited with the community overcoming a cultural aversion to outsiders and skepticism about their motives. In fact, the community reached out to the EM team for assistance in addressing the opioid issue and breaking down existing social barriers to action. The community had a good deal of trust that the EM team had their best interests in mind.

We had a great community presence there because we had done a community needs assessment already with a local coordinator and she continued working with us as the coordinator with the lung cancer project that had some wonderful outcomes. Then the community came back to us and said you know we’ve got this opioid problem, and we’d like to apply SEED to that so then we just kept going so it’s been a while.

So over time, with being more personable, and understanding each other trust has grown, to the point where I don’t think that anything on the academic side is ill-willed or malicious, and I don’t think on the community side either.

And, because we’ve been doing this for so long, the trust is now based on experience versus based on some assumptions.

### USING A STRUCTURED METHOD OF ENGAGEMENT

Team members credited the partnership processes that are part of the SEED Method with helping to develop trust and allowing them to feel safe to express themselves without judgment, put aside their own biases, and work together. They felt the SEED process provided the safety and structure to build trust between individuals and institutions.

Although the partners came from varying backgrounds, responses to the survey showed that the process was able to bridge that gap to unify the partners around a core understanding and vision for the project (core values mean score = 6.35/7, s.d. 0.21 – Table 3). This may have been accomplished through the effective implementation of the principles of community engagement that are part of the SEED Method as indicated by the survey findings of significant alignment with principles of community engagement (4.82/6, s.d. 0.21) (Table 3).

Interviewees highlighted the importance of a structured process that moved partners from research to action and credited the process with breaking down barriers to collaboration to accomplish goals. They identified the accomplishments of the four work groups on the prioritised actions as the most important outcomes of the project, and credited the process for getting the community working together. Less tangible outcomes included the impact of the project on networking within the community, and the capacity of the community to approach a problem and set achievable goals.

Reminds me of like a mediator, not that they were mediating. But you know, it was just having that third party with a structure and a framework, and a goal, and very specific tasks to keep us on track, you know, track and focus.

They have been trying to get this [community strategy] done. Why did it happen? I think it was just a result of going through this process and getting enough people sitting at the table and having the community say we want this and we’re going to support it, that then was able to drive it forward.

We still work for the [public agency], but it’s amazing, we have a partnership with a private hospital, and we are a public community service. This has never happened!

We are not the silos to the extent that we were, I really don’t think so. I think that’s the greatest success (of the project), that we acknowledge that we need each other.

The team also attributed the breakdown of interpersonal barriers between team members and other stakeholders to the CBPR principles of shared power, mutual respect and shared decision making that are foundational to the SEED Method.

I think the SEED method is just a great best practice as far as trying to eliminate from one person dominating the outcomes, because the whole process is used to weed that out, because everyone has to participate.

I was astounded because [NAME 2] and [NAME 3] and the way the SEED method is set up, provided this platform that was safe, without interference of personal bias… they did just such a great job keeping things neutral. And when that environment was established, then the partnership of trust could really blossom.

### OUTCOMES FROM THE RIPPLE EFFECTS MAPPING

**Figure 3.**
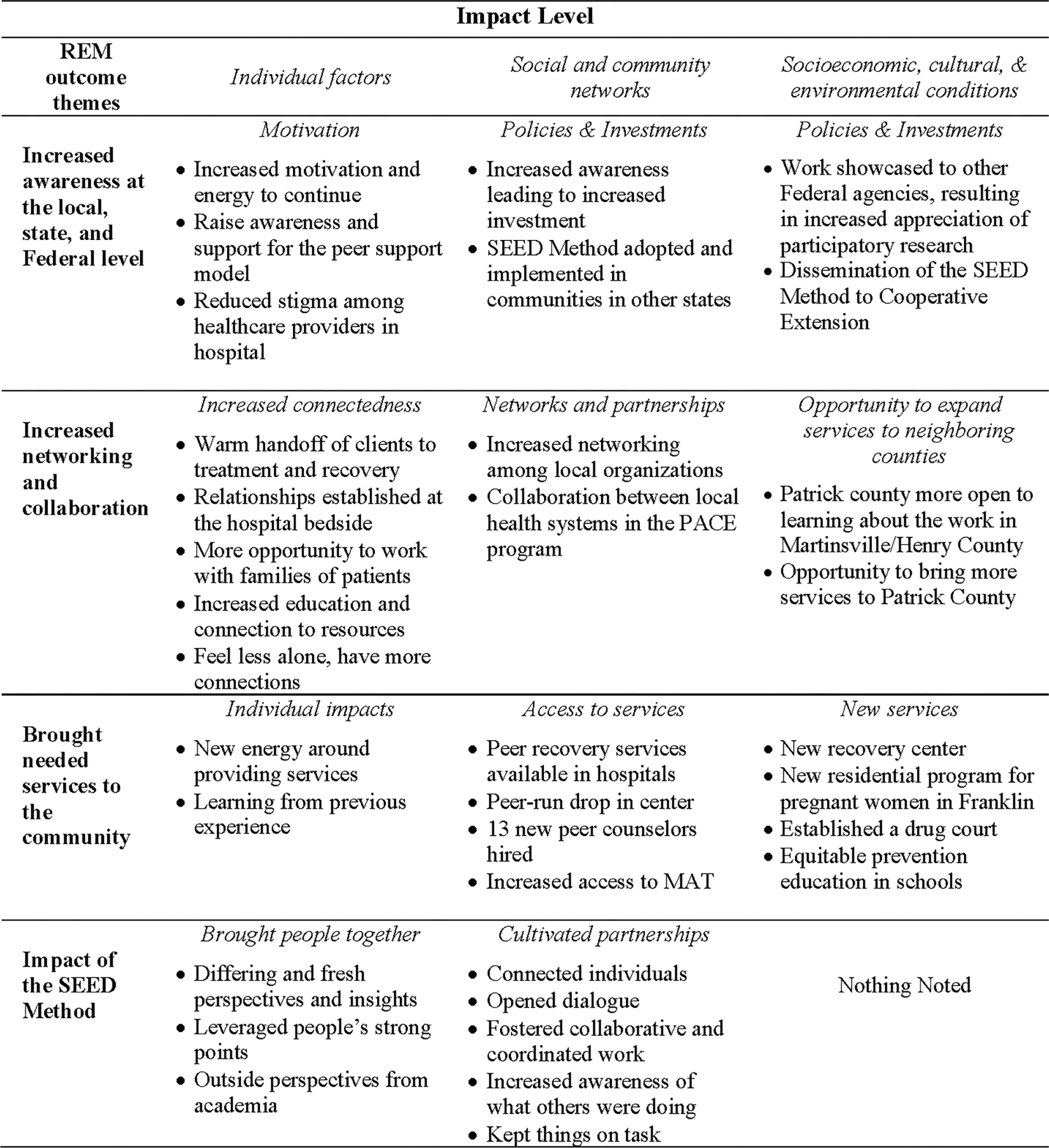
Ripple Effects Mapping (REM) results

In the Ripple Effects Mapping exercise, participants described project outcomes that increased community awareness, improved collaboration between community organizations, particularly the physical and mental healthcare organizations, and increased the availability of local services (Figure 3). They described how these outcomes resulted in reduced stigma, increased community connectedness and understanding, and reduced isolation for those impacted by substance misuse. Networking and collaboration improved between community organizations, particularly the physical and mental healthcare organizations, resulting in systems for ensuring a continuum of care for individuals with substance use disorder (SUD), expansion of peer recovery programs and access to medically assisted treatment (MAT). Team members credited the systematic approach of the SEED Method with fostering this collaboration through open dialogue, coordination of effort and sustained engagement (Figure 3).

Finally, presentations by the team increased awareness of key agencies that could support local responses to socioeconomic, cultural and environmental conditions. Federal and state funds supported the establishment of a new recovery and residential center, a regional recovery court and prevention education that served the region. Networking across county lines increased the readiness of adjacent counties to address the opioid and substance misuse issue and afforded the opportunity to bring services to those communities.

## Discussion

Evaluation of community engagement is a topic of much research, as it is essential to bridge the gap between science and practice. Models have been created to define meaningful engagement and assess its impact (Khodjakov et al. 2011; Organizing Committee 2022; Wallerstein et al. 2008). These models define the key domains of community engagement principles with indicators of impact when applied effectively (Organizing Committee 2022). The diversity of context in which community engagement occurs has necessitated that these models be flexible and provide a range of outcome options. Specific common metrics that can be used across community-engaged research projects are still needed.

This project serves as a case study for evaluating community engagement practices. Using the CBPR model and tools, we were able to evaluate the relative success of our community-engaged project in relationship building and developing trust within our community-university partnership, the ability to create significant changes benefitting the community, and the impact of the SEED Method on these outcomes. By evaluating the various domains within the CBPR model (i.e., context, partnership processes, intervention and research and outcomes) we were able to identify factors that facilitated the project’s perceived personal and community benefits. We identified several factors that helped to facilitate positive outcomes. The partnership benefitted from an existing level of trust based on returning to work with the community following a previous project. Another facilitator was having a clearly defined health issue that was important to the community and created a common vision and focus for the project. The effective implementation of processes that established quality and equitable relationships among partnership members was a key factor.

Trust was a prominent theme in our analysis. Trust is known to strengthen partnership synergy and sustainability and is necessary for population-level outcomes such as systemic transformations, health improvement and spin-off projects (Jagosh et al. 2015; Lasker, Weiss & Miller 2001). Development of trust was facilitated by processes that promoted mutual respect, open dialogue and participatory decision making, as well as the long-term engagement and completion of the project objectives by the academic partners (Jagosh et al. 2015; Lucero 2013; Peralta, Smithwick & Torres 2020).

We used a mixed-methods approach to assess the outcomes of the project. These included gathering the perspectives of project participants through surveys and qualitative interviews based on the CBPR model, documentation of the outcomes of the work groups formed to implement prioritised strategies recommended by the community, and a Ripple Effects Mapping Exercise (REM) that allowed community members to reflect on the positive, negative and unintended impacts of the project. Despite the small number of participants, there was concurrence on the characteristics of the partnership as well as the tangible outcomes of the project between project participants that was evident in all three of the evaluation methods. Participants were excited about the tangible accomplishments of the work groups, and also enthusiastically described the importance of relationship building, trust and using a structured process. REM allowed for an exploration of unintended project outcomes. In particular, REM identified a reduction in stigma among providers and community residents that resulted in increased openness to others’ perspectives and a decrease in the isolation felt by many individuals and family members dealing with SUD.

The use of a structured method for engaging and directing community activities was credited with breaking down long-standing silos between community organizations and mobilizing the community for action. It was also credited with facilitating positive working relationships and building individuals’ skills and self-confidence. The SEED Method seeks to address key determinants identified as important to partnership synergy by Lasker, Weiss and Miller (2001) including the equitable use of resources, diverse stakeholder engagement in the partnership, cultivation of trust and mutual respect and a planned process for partnership administration leading to community action. Interview respondents indicated that the SEED Method provided a safe space where participants could get to know each other, overcome biases and learn to work together for the community good. Effective leaders were credited with facilitating this safe environment.

This study has limitations including the small sample size and the lack of participation in the interviews of community research team members who left before the end of the project. In addition, although the importance of relationship building, trust and a planned and structured method of engagement has broad application, evaluation results are specific to this project’s context which may limit generalizability.

Partnership structure impacts partner processes and synergy (Lasker, Weiss & Miller 2001; Weiss et al. 2002), which in turn impacts community-level outcomes (Khodjakov et al. 2011). Our evaluation confirmed satisfaction with partnership function and a high degree of partnership synergy, which undoubtedly contributed to the personal and community level outcomes. More recent research is attempting to determine the partnering practices within specific contexts that effectively contribute to research, community and health equity outcomes (Wallerstein at al. 2020). This case study provides a detailed description of the context of a community engaged project that followed a defined method grounded in the core principles of community engagement, as well as the tangible and perceived personal and community level outcomes identified through a mixed-methods evaluation approach. Although not evaluated in this study, we will incorporate measures of health equity outcomes in future projects. Our findings may inform the approach to engagement of other community-based participatory research projects, as well as their evaluation approach.

## Figures and Tables

**Table 1.** CBPR Survey – Relationships

Relationships	Average Score	SD	Scale Mean Scores

Quality of Dialogue (7-point scale)*			
We show positive attitudes towards one another.	6.25	0.71	5.63 (1.21)
Everyone in our partnership participates in our meetings.	5.13	1.46	
We listen to each other.	6.25	0.89	
When conflicts occur, we work together to resolve them.	6.25	0.71	
Even when we don’t have total agreement, we reach a kind of consensus that we all accept.	6.13	0.99	
The dialogue is dominated by the perspectives of the academic partners.	3.25	1.16	
Participatory Decision Making (7-point scale)*			
Suggestions I make in the partnership are seriously considered.	5.38	1.41	5.07 (0.24)
I have influence over decisions.	4.88	1.46	
My involvement influences the partnership to be more responsive to community.	5.13	0.83	
I am able to influence the work of the project.	4.88	1.36	
Leadership (6-point scale)**			
Encourage active participation of academic and community partners in decision making	5.25	0.71	5.10 (0.37)
Communicate goals of the project	5.38	0.52	
Resolve conflict among partners	4.50	1.07	
Foster respect between partners	5.38	0.74	
Help partners be creative and look at things differently	5.00	1.07	

* 1=completely disagree, 2=mostly disagree, 3=slightly disagree, 4=neither agree nor disagree, 5=slightly agree, 6=mostly agree, 7=completely agree

** 1=not at all well, 2=somewhat well, 3=moderately well, 4=very well, 5=extremely well, 6=completely well

**Table 2.** CBPR Survey – Trust Scale

Trust (7-point scale)*	Average Score	SD	Scale Mean Score
I trust the decisions others make about issues important to our project.	6.13	0.83	6.29 (0.19)
I can rely on the people that I work with on this project.	6.25	0.71	
People in this partnership have a lot of confidence in one another.	6.50	0.53	

* 1=completely disagree, 2=mostly disagree, 3=slightly disagree, 4=neither agree nor disagree, 5=slightly agree, 6=mostly agree, 7=completely agree

**Table 3.** CBPR Survey - Partnership Core Values and Community Engagement Principles Scale

Partnership Core Values (7-point scale)**	Average Score (s.d)	SD	Scale Mean Score (s.d)
Members have clear and shared understanding of the problems	6.63	0.52	6.35 (0.21)
Members can generally state the partnership mission and goals	6.13	0.64	
General agreement on priorities of the partnership	6.25	0.71	
General agreement on the strategies partnership should use in pursuing priorities	6.38	0.52	
Alignment with Community Engagement Principles (6-point scale)*	Average Score (s.d)	SD	Scale Mean Score (s.d)
Project builds resources and strengths in the community	4.88	0.64	4.82 (0.21)
Project facilitates equitable partnerships in all phases	4.63	0.92	
Project helps all partners to grow and learn from one another	4.88	0.99	
Project balances research and social action for mutual benefit of all partners	5.13	0.83	
Project emphasises factors that are important to the community that affect well-being	4.88	0.99	
Project communicates knowledge and findings, and involves all partners in the dissemination	5.13	0.64	
Project views CBPR as a long-term process and commitment	4.75	1.04	
Project is responsive to community histories	4.50	0.93	
Project integrates the words and language of the community	4.63	0.74	
Project connects with the way things are done in the community	4.75	0.71	

* 1=not at all, 2=to a small extent, 3=to a moderate extent, 4 = to a great extent, 5 = to a very great extent, 6 = to a complete extent

## APPENDIX 1 Engaging Martinsville Partner Survey

Thank you for taking this survey! This survey is part of an ongoing project, Engaging Martinsville & Henry County: Community action on the opioid crisis. This project aims to bring residents of Martinsville and Henry County together to take action to address the opioid issue in the community.

The terms *project, partnership*, and *partner* are used in this survey. *Project* refers to the research project you have been a part of: Engaging Martinsville. *Partnership* refers to a collaboration of partners working together for a common goal of which Engaging Martinsville may be only one part. *Partners* include both community partners and academic partners.

Community partners represent perspectives and knowledge of communities and can be individuals (such as community members, patients, caregivers, clinical providers, healthcare staff, policy makers, or other individual stakeholders) or organizations (such as community based organizations, advocacy organizations, agencies, tribal programs, clinics, health department, or other groups representing communities).

Academic partners represent research knowledge and are individuals and organizations associated with universities, or other institutions that house research.

Thank you for your time and attention!

Q1 What was your primary role in the Engaging Martinsville opioid project? Please select one response.

- Academic research team member (1)
- Topic group member (2)
- Work group member (3)

Q2 Select the most appropriate response.

**Table:**

	Not at all (1)	To a small extent (2)	To a moderate extent (3)	To a great extent (4)	To a very great extent (5)	To a complete extent (6)	I don’t know (7)
The community participating in this project has a history of organizing services or events. (1)	○	○	○	○	○	○	○
The community participating in this project has a history of advocating for social or health equity. (2)	○	○	○	○	○	○	○
By working together, people in the community participating in this project have previously influenced decisions that affected their communities. (3)	○	○	○	○	○	○	○

Q3 Does this partnership (including Engaging Martinsville, community partner organizations, and work group participants) have any of the following features to achieve the project aims of implementing community identified strategies to address opioid misuse in MHC.

**Table:**

	Not at all (1)	To a small extent (2)	To a moderate extent (3)	To a great extent (4)	To a very great extent (5)	To a complete extent (6)
Skills and expertise (1)	○	○	○	○	○	○
Diverse members (2)	○	○	○	○	○	○
Legitimacy and credibility in the community (3)	○	○	○	○	○	○
Ability to bring people together for meetings/ activities (4)	○	○	○	○	○	○
Connections to relevant stakeholders (5)	○	○	○	○	○	○

Q4 Please choose the most appropriate response from your perspective.

**Table:**

	Not at all (1)	To a small extent (2)	To a moderate extent (3)	To a great extent (4)	To a very great extent (5)	To a complete extent (6)
The community partners (such as community members or organizations) have the knowledge, skills, and confidence to interact effectively with the academic partners. (1)	○	○	○	○	○	○
The academic partners have members who are from a similar background as the community partners. (2)	○	○	○	○	○	○
The academic partners have the knowledge, skills, and confidence to interact effectively with the community partners. (3)	○	○	○	○	○	○

Q5 Please choose the most appropriate response from your perspective.

**Table:**

	Completely disagree (1)	Mostly disagree (2)	Slightly disagree (3)	Neither agree nor disagree (4)	Slightly agree (5)	Mostly agree (6)	Completely agree (7)
Members of our partnership have a clear and shared understanding of the problems we are trying to address. (1)	○	○	○	○	○	○	○
Members can generally state the mission and goals of our partnership. (2)	○	○	○	○	○	○	○
There is general agreement with respect to the priorities of our partnership. (3)	○	○	○	○	○	○	○
There is general agreement on the strategies our partnership should use in pursuing its priorities. (4)	○	○	○	○	○	○	○

Q6 How much have community partners been involved in the following activities?

**Table:**

	Not at all involved (1)	Somewhat involved (2)	Moderately involved (3)	Very involved (4)	Extremely involved (5)	Completely involved (6)
Informing the community about research progress and findings. (1)	○	○	○	○	○	○
Informing relevant policy makers about findings. (2)	○	○	○	○	○	○
Sharing findings with other communities. (3)	○	○	○	○	○	○
Producing useful findings for community action and benefit. (4)	○	○	○	○	○	○

Q7 Thinking about your involvement in the work group and/or larger project, mark the most appropriate response.

**Table:**

	Completely disagree (1)	Mostly disagree (2)	Slightly disagree (3)	Neither agree nor disagree (4)	Slightly agree (5)	Mostly agree (6)
Suggestions I make within this partnership are seriously considered. (1)	○	○	○	○	○	○
I have influence over decisions that this partnership makes. (2)	○	○	○	○	○	○
My involvement influences the partnership to be more responsive to the community. (3)	○	○	○	○	○	○
I am able to influence the work on this project. (4)	○	○	○	○	○	○

Q8 How much do you agree or disagree that this partnership has conversations where:

**Table:**

	Completely disagree (1)	Mostly disagree (2)	Slightly disagree (3)	Neither agree nor disagree (4)	Slightly agree (5)	Mostly agree (6)	Completely agree (7)
We show positive attitudes towards one another. (1)	○	○	○	○	○	○	○
Everyone in our partnership participates in our meetings. (2)	○	○	○	○	○	○	○
We listen to each other. (3)	○	○	○	○	○	○	○
When conflicts occur, we work together to resolve them. (4)	○	○	○	○	○	○	○
Even when we don’t have total agreement, we reach a kind of consensus that we all accept. (5)	○	○	○	○	○	○	○
The dialogue is dominated by the perspectives of the academic partners. (6)	○	○	○	○	○	○	○

Q9 How well does the leadership for the partnership:

**Table:**

	Not at all well (1)	Somewhat well (2)	Moderately well (3)	Very well (4)	Extremely well (5)	Completely well (6)
Encourage active participation of academic and community partners in decision making. (1)	○	○	○	○	○	○
Communicate the goals of the project. (2)	○	○	○	○	○	○
Resolve conflict among partners. (3)	○	○	○	○	○	○
Foster respect between partners. (4)	○	○	○	○	○	○
Help the partners be creative and look at things differently. (5)	○	○	○	○	○	○

Q10 How well does your project use:

**Table:**

	Not at all well (1)	Somewhat well (2)	Moderately well (3)	Very Well (4)	Extremely well (5)	Completely well (6)
The partnership’s financial resources. (1)	○	○	○	○	○	○
The partnership’s in-kind resources. (2)	○	○	○	○	○	○
The partners’ time. (3)	○	○	○	○	○	○

Q11 How much do you agree or disagree with these statements about the level of trust between the partnership members.

**Table:**

	Completely disagree (1)	Mostly disagree (2)	Slightly disagree (3)	Neither agree nor disagree (4)	Slightly agree (5)	Mostly agree (6)	Completely agree (7)
I trust the decisions others make about issues that are important to our project. (1)	○	○	○	○	○	○	○
I can rely on the people that I work with on this project. (2)	○	○	○	○	○	○	○
People in this partnership have a lot of confidence in one another. (3)	○	○	○	○	○	○	○

Q12 Does this project reflect the following Community Based Participatory Research (CBPR) principles?

**Table:**

	Not at all (1)	To a small extent (2)	To a moderate extent (3)	To a moderate extent (4)	To a great extent (5)	To a very great extent (6)	To a complete extent (7)
This project builds on resources and strengths in the community. (1)	○	○	○	○	○	○	○
This project facilitates equitable partnerships in all phases of the research. (2)	○	○	○	○	○	○	○
This project helps all partners involved to grow and learn from research. (3)	○	○	○	○	○	○	○
This project balances research and social action for the mutual benefit of all partners. (4)	○	○	○	○	○	○	○
This project emphasizes the factors that are important to the community (e.g., environmental and social factors) which affect well-being. (5)	○	○	○	○	○	○	○
This project communicates knowledge and findings to all partners and involves all partners in the dissemination process. (6)	○	○	○	○	○	○	○
This project views CBPR or community engaged research as a long-term process and a long-term commitment. (7)	○	○	○	○	○	○	○
This project is responsive to community histories. (8)	○	○	○	○	○	○	○
This project integrates the words and language of the community. (9)	○	○	○	○	○	○	○
This project connects with the ways things are done in the community. (10)	○	○	○	○	○	○	○

Q13 How much do or will the community or clinical organizations in this partnership enjoy the following benefits?

**Table:**

	Not at all (1)	To a small extent (2)	To a moderate extent (3)	To a great extent (4)	To a very great extent (5)	To a complete extent (6)
Enhanced reputation. (1)	○	○	○	○	○	○
Enhanced ability to affect public policy. (2)	○	○	○	○	○	○
Increased use of the agency’s expertise or services by others. (3)	○	○	○	○	○	○

Q14 Do you experience the following difficulties related to participating in this partnership?

**Table:**

	Not at all (1)	To a small extent (2)	To a moderate extent (3)	To a great extent (4)	To a very great extent (5)	To a complete extent (6)
Negative views from outside of the partnership of your participation in the partnership. (1)	○	○	○	○	○	○
Frustration with the amount of time and resources spent for the outcomes achieved. (2)	○	○	○	○	○	○
Time or resources taken away from other activities you value. (3)	○	○	○	○	○	○

Q15 How much do you agree or disagree that:

**Table:**

	Completely disagree (1)	Mostly disagree (2)	Slightly disagree (3)	Neither agree nor disagree (4)	Slightly agree (5)	Mostly agree (6)	Completely agree (7)
I am committed to sustaining the community-academic relationship with no or low funding. (1)	○	○	○	○	○	○	○
This project is likely to continue forward after this funding is over. (2)	○	○	○	○	○	○	○
Our partnership carefully evaluates funding opportunities to make sure they meet both community and academic partners’ needs. (3)	○	○	○	○	○	○	○

Q16 Provide the response that best represents your opinion.

**Table:**

	Not at all (1)	To a small extent (2)	To a moderate extent (3)	To a great extent (4)	To a very great extent (5)	To a complete extent (6)
How much do you think this project will improve the health of the community? (1)	○	○	○	○	○	○
How much do you think this project will improve the health behaviors of community? (2)	○	○	○	○	○	○

Q17 Please provide the most appropriate response to the questions about the quality of the work and your satisfaction with it.

**Table:**

	Not at all good (1)	Somewhat good (2)	Moderately good (3)	Very good (4)	Extremely good (5)	Completely good (6)
What is the quality of the overall work of the partnership toward achieving the goals of the project? (1)	○	○	○	○	○	○
How satisfied are you with your partnering experience on this project? (2)	○	○	○	○	○	○

Q18 What has been the most important outcome of this project?

________________________________________________________________________________

Q19 Can you tell us anything else about positive or negative outcomes not captured in this survey?

________________________________________________________________________________

## APPENDIX 2 Interview guide for self-reflection and evaluation of CBPR partnerships: Version, 2017

Adapted from Research for Improved Health: A Study of Community-Academic Partnerships

### I. Introduction

Thank you so much for agreeing to help us evaluate your partnership. You are being asked to participate because you are a valued member and partner. The purpose of these interviews is to facilitate a reflection and self-evaluation of the partnership by its members. We are asking these questions to learn about your experiences, so that we can better understand how the partnership is doing, and how it can be improved to better meet its mission of promoting health and well-being within the community.

This partnership is very invested in learning from the experiences of its members. The quality of an evaluation like this relies on participants’ openness and willingness to share their experiences, both positive and negative.

We value your insight and expertise, so we’d like you to share, in your own words, the successes, any challenges, as well as any outcomes that you feel may have come from this partnership. The data from these interviews will be analyzed by the evaluation partners at Virginia Tech. The partnership will receive the overall results and will not see individual interview responses.

### II. Individual Background *(keep this section brief)*

First, we are going to talk a bit about you and your involvement in the partnership.

1. Tell me how you came to be involved in Engaging Martinsville. What motivated you to start working with the partnership?
2. Are you representing an organization in the partnership or are you participating as yourself? If representing an organization, which one?
3. Tell me about your role and the work you are doing in the partnership? (keep brief)

### III. Context for All Communities

Next, we will talk specifically about your community and/or your organization’s partnership with Engaging Martinsville.

1. (If you are representing an organization): In thinking of your organization and its participation in the partnership, what kinds of strengths, assets, skills and/or resources do you think your organization brings to the partnership? Probe: Can you provide an example(s)?
2. (If you are representing an organization): What challenges or limitations did your organization have when you first joined the partnership? Probe: Can you provide an example(s)?
3. What should we know about your community (its strengths or challenges) to help us better understand its involvement with the partnership? Probe: Can you provide an example(s)?
4. How do you think (*academic partner*) was perceived in the community when you started with the partnership? How do you think it is perceived now?

### IV. Partnership/Group Dynamics

Next, we are going to talk a bit more about how the partnership works together as a group.

1. Can you describe how members of the partnership work together? What has gone well in your meetings and in getting your tasks done and what have been the challenges?
2. Given the diversity represented within the partnership, how are membership needs and interests met? For example, when a group of people with such diverse backgrounds meets, how are differences managed to enable the partnership to move forward?
3. Trust is a necessary part of partnerships. Please share an example of a situation that ***strengthened trust*** within the partnership?
4. How about an example of a situation that ***challenged trust*** within the partnership?
5. Think back to when you began your involvement with the partnership. What did trust look like then versus now? Has it changed? How?
6. Tell me about the leadership within the partnership. Who are the leaders?
7. In terms of leadership styles or approaches, what’s worked well? (Probes: in terms of how partnership resources have been managed, or guiding the direction, decision-making, etc.)
8. What has not worked as well?
9. (If this hasn’t come up earlier): How are decisions about partnership issues made within Engaging Martinsville?
10. What works well in the decision-making process?
11. What’s not worked as well?
12. (IF NOT ALREADY ADDRESSED): Can you talk a little about how you deal with conflict in the group? What has worked well? What’s not working well?
13. We are interested in how communication works among the membership. Tell me how communication works in the group. For example, if you wanted to raise an issue or influence the direction the partnership takes related to a project, how would you go about that?
14. Who are the people in the partnership that you have the most contact with?
15. Thinking about everything we just talked about, what could be done to improve how the partnership functions and works together?
16. What do you see as your role in these changes?

### V: Intervention & Research Design

Since _____ research projects have focused on interventions, next we will talk about the process of developing and implementing these projects or interventions.

Which projects have you been involved in?

Let’s talk about your experience with one of the projects, whichever one you choose, or feel you know best. Which one would you like to talk about? ________

1. In what ways did knowledge and experience from the community influence the project?
2. In what ways has knowledge from previous research, ‘evidence’ and ‘best practices’ from around the country influenced your project/intervention?
3. Think about how community members and/or community-based organizations have been involved in research processes. Tell me about the role they play in each step of the process, i.e., from proposing the research questions to research design and intervention. (For example, recruitment, data collection, analysis and interpretation?)
4. How have project findings been shared with the community? What other ideas do you have for communicating results?
5. How do you think using a CBPR approach has influenced the work of the partnership towards achieving the project’s goals?

### VI. Intervention and Policy Outcomes

Next, we will talk about outcomes of the project we have been discussing and of the partnership in general.

1. Staying with the project now, what outcomes or benefits have you seen or do you expect to see as a result of the intervention?
2. Do you think the community is aware of the benefits or intended benefits of the project? How?
3. Do you think this project has contributed to any practice or policy changes at the community level? If so, how? What strategies were used to promote this change? (i.e. presenting data, telling stories)

For the rest of the interview, we will switch back to discussing the partnership as whole, rather than the individual project.

1. (If you are an organizational partner): How has participation in the partnership changed your organization? (i.e. new capacities or skills? New practices or policies?)
2. (If you are a university partner): How has the partnership changed the way the university does business? (i.e. any of its policies or practices in doing research with communities?)
3. As an individual, how have you benefitted from being a member of the partnership?
4. How has the partnership impacted the health status of the community? What are some specific examples?

### VII. Partnership Outcomes

We now want to dig a little deeper into partnership outcomes that you are hoping to achieve through your work with Engaging Martinsville.

1. What would you say have been the most important successes for the partnership?
2. Like many CBPR partnerships, Engaging Martinsville relies heavily on individuals and organizations volunteering their time and resources. Where do you think things stand in terms of sustainability for the partnership?
3. What would make the partnership more sustainable?

### VIII. Summary

We’re coming to the end now, and I have just two more questions for you.

1. If another group were going to start this kind of partnership, what kinds of things would you tell them in order to help them be successful?
2. Is there anything else you’d like to add?

We would like to express our sincerest gratitude for sharing your thoughts and experiences with us here today. Your time and devotion are truly appreciated, respected and matter. Many thanks!!

## References

[R1] Belone L, Lucero JE, Duran B, Tafoya G, Baker EA, Chan D, Chang C, Greene-Moton E, Kelley MA & Wallerstein N 2016, ‘Community-based rarticipatory research conceptual model: Community partner consultation and face validity’, Qualitative Health Research, vol. 26, no. 1, pp. 117–35. 10.1177/1049732314557084 25361792 PMC4839192

[R2] Boursaw B, Oetzel JG, Dickson E, Thein TS, Sanchez-Youngman S, Peña J, Parker M, Magarati M, Littledeer L, Duran B & Wallerstein N 2021, ‘Scales of practices and outcomes for community-engaged research’, American Journal of Community Psychology, vol. 67, no. 3–4, pp. 256–70. 10.1002/ajcp.12503 33599288 PMC8355013

[R3] Chazdon S, Emery M, Hansen D, Higgins L & Sero R 2017, A field guide to ripple effects mapping, University of Minnesota Libraries Publishing.

[R4] Childs E, Biello KB, Valente PK, Salhaney P, Biancarelli DL, Olson J, Earlywine J, Marshall BDL & Bazzi AR 2021, ‘Implementing harm reduction in non-urban communities affected by opioids and polysubstance use: A qualitative study exploring challenges and mitigating strategies’, The International Journal on Drug Policy, vol. 90, 103080. 10.1016/j.drugpo.2020.103080 33340947 PMC8046716

[R5] Dedding C, Goedhart NS, Broerse JE & Abma TA 2021, ‘Exploring the boundaries of “good” participatory action research in times of increasing popularity: Dealing with constraints in local policy for digital inclusion’, Educational Action Research, vol. 29, no. 1, pp. 20–36. 10.1080/09650792.2020.1743733

[R6] Emery M, Higgins L, Chazdon S & Hansen D 2015, ‘Using ripple effect mapping to evaluate program impact: Choosing or combining the methods that work best for you’, Journal of Extension, vol. 53, no. 2. 10.34068/joe.53.02.36

[R7] Engage for Equity, n.d., Expanding Community Engaged Surveys to More Partners, viewed 2 January 2021, https://engageforequity.org/tool_kit/surveys/.

[R8] Gale N, Heath G, Cameron E, Rashid S & Redwood S 2013, ‘Using the framework method for the analysis of qualitative data in multi-disciplinary health research’, BMC Medical Research Methodology, vol. 13, p. 117. 10.1186/1471-2288-13-117 24047204 PMC3848812

[R9] Hargrove AJ, Rafie C, Zimmerman E & Moser DE 2022, ‘A rural community’s perspective on the causes of and solutions to the opioid crisis in southern Virginia: A qualitative study’, Rural Remote Health, June, vol. 22, no. 2, p. 7152. Epub 2022 Jun 1. 10.22605/RRH7152 35641244 PMC9310544

[R10] HHS Press Office 2017, HHS Acting Secretary declares public health emergency to address national opioid crisis, news release, U.S. Department of Health and Human Services, 26 October. https://www.hhs.gov/about/news/2017/10/26/hhs-acting-secretary-declares-public-health-emergency-address-national-opioid-crisis.html

[R11] Hoffman A, Montgomery R, Aubry W & Tunis SR 2010, ‘How best to engage patients, doctors, and other stakeholders in designing comparative effectiveness studies’, Health Affairs (Millwood), vol. 29, no. 10, pp. 1834–41. 10.1377/hlthaff.2010.0675 20921483

[R12] Höppner C 2009, ‘Trust—A monolithic panacea in land use planning?’, Land Use Policy, vol. 26, no. 4, pp. 1046–54. 10.1016/j.landusepol.2008.12.007

[R13] Israel BA, Coombe CM, Cheezum RR, Schulz AJ, McGranaghan RJ, Lichtenstein R, Reyes AG, Clement J & Burris A 2010, ‘Community-based participatory research: A capacity-building approach for policy advocacy aimed at eliminating health disparities’, American Journal of Public Health, vol. 100, no. 11, pp. 2094–102. 10.2105/AJPH.2009.170506 20864728 PMC2951933

[R14] Israel BA, Lachance L, Coombe CM, Lee SD, Jensen M, Wilson-Powers E, Mentz G, Muhammad M, Rowe Z, Reyes AG & Brush BL 2020, ‘Measurement approaches to partnership success: Theory and methods for measuring success in long-standing community-based participatory research partnerships’, Progress in Community Health Partnerships: Research, Education, and Action, vol. 14, no. 1, pp. 129–40. 10.1353/cpr.2020.0015 32280130 PMC7439287

[R15] Jagosh J, Bush PL, Salsberg J, Macaulay AC, Greenhalgh T, Wong G, Cargo M, Green LW, Herbert CP & Pluye P 2015, ‘A realist evaluation of community-based participatory research: Partnership synergy, trust building and related ripple effects’, BMC Public Health, vol. 15, p. 725. 10.1186/s12889-015-1949-1 26223523 PMC4520009

[R16] Jagosh J, Macaulay AC, Pluye P, Salsberg J, Bush PL, Henderson J, Sirett E, Wong G, Cargo M, Herbert CP, Seifer SD, Green LW & Greenhalgh T 2012, ‘Uncovering the benefits of participatory research: Implications of a realist review for health research and practice’, The Milbank Quarterly, vol. 2, no. 90, pp. 311–46. 10.1111/j.1468-0009.2012.00665.x PMC3460206 22709390

[R17] Kapiriri L & Norheim OF 2002, ‘Whose priorities count? Comparison of community-identified health problems and burden-of-disease assessed health priorities in a district in Uganda’, Health Expectations, vol. 5, no. 1, pp. 55–62. 10.1046/j.1369-6513.2002.00161.x 11915845 PMC5060125

[R18] Khodyakov D, Stockdale S, Jones F, Ohito E, Jones A, Lizaola E & Mango J 2011, ‘An exploration of the effect of community engagement in research on perceived outcomes of partnered mental health services projects’, Society and Mental Health, vol. 1, no. 3, pp. 185–99. 10.1177/2156869311431613 22582144 PMC3349344

[R19] Lasker RD, Weiss ES & Miller R 2001, ‘Partnership synergy: A practical framework for studying and strengthening the collaborative advantage’, The Milbank Quarterly, vol. 79, no. 2, pp. 179–204. 10.1111/1468-0009.00203 11439464 PMC2751192

[R20] Lomas J, Fulop N, Gagnon D & Allen P 2013, ‘On being a good listener: Setting priorities for applied health services research’, The Milbank Quarterly, vol. 81, no. 3, pp. 363–88. 10.1111/1468-0009.t01-1-00060 PMC2690239 12941000

[R21] Lucero JE 2013, ‘Trust as an ethical construct in community based participatory research partnerships’. https://digitalrepository.unm.edu/cj_etds/43

[R22] Luger TM, Hamilton B & True G 2020, ‘Measuring community-engaged research contexts, processes, and outcomes: A mapping review’, The Milbank Quarterly, vol. 98, no. 2, pp. 493–553. 10.1111/1468-0009.12458 32428339 PMC7296434

[R23] McElfish PA, Kohler P, Smith C, Warmack S, Buron B, Hudson J, Bridges M, Purvis R & Rubon-Chutaro J 2015, ‘Community-driven research agenda to reduce health disparities’, Clinical and Translational Science, vol. 8, no 6, pp. 690–95. 10.1111/cts.12350 26573096 PMC4703475

[R24] Meyer J 2000, ‘Evaluating action research’, Age and Ageing, vol. 29, suppl 2, pp. 8–10. 10.1093/oxfordjournals.ageing.a008104 11109939

[R25] Oetzel JG, Zhou C, Duran B, Pearson C, Magarati M, Lucero J, Wallerstein N & Villegas M 2015, ‘Establishing the psychometric properties of constructs in a community-based participatory research conceptual model’, American Journal of Health Promotion, vol. 29, pp. e188–e202. 10.4278/ajhp.130731-QUAN-398 24720389 PMC4819435

[R26] Oetzel JG, Wallerstein N, Duran B, Sanchez-Youngman S, Nguyen T, Woo K, Wang J, Schulz A, Keawe’aimoku Kaholokula J, Israel B & Alegria M 2018, ‘Impact of participatory health research: A test of the community-based participatory research conceptual model’, BioMed Research International, 7281405. 10.1155/2018/7281405 29854784 PMC5941804

[R27] O’Haire C, McPheeters M, Nakamoto EK, LaBrant L, Most C, Lee K, Graham E, Cottrell E & Guise JM 2011, Engaging stakeholders to identify and prioritise future research needs, Methods Future Research Needs Report Number 4, Agency for Healthcare Research and Quality. 21977526

[R28] Oliver S, Clarke-Jones L, Rees R, Milne R, Buchanan P, Gabbay J, Gyte G, Oakley A & Stein K 2004, ‘Involving consumers in research and development agenda setting for the NHS: Developing an evidence-based approach’, Health Technology Assessment, vol. 8, no. 15, pp. 1–148. 10.3310/hta8150 15080866

[R29] Organizing Committee for Assessing Meaningful Community Engagement in Health & Health Care Programs & Policies 2022, Assessing meaningful community engagement: A conceptual model to advance health equity through transformed systems for health, NAM Perspectives, Commentary, National Academy of Medicine, Washington, DC. 10.31478/202202c PMC9303007 35891775

[R30] Peralta AM, Smithwick J & Torres ME 2020, ‘Perceptions and determinants of partnership trust in the context of community-based participatory research’, Journal of Health Disparities Research and Practice, vol. 13, no. 1, pp. 67–95. https://digitalscholarship.unlv.edu/jhdrp/vol13/iss1/4

[R31] Pohl C & Hadorn GH 2008, ‘Methodological challenges of transdisciplinary research’, Natures Sciences Sociétés, vol. 16, no. 2, pp. 111–21. 10.1051/nss:2008035

[R32] Rafie C, Zimmerman EB, Moser DE, Cook S & Zarghami F 2019, ‘A lung cancer research agenda that reflects the diverse perspectives of community stakeholders: Process and outcomes of the SEED method’, Research Involvement and Engagement, vol. 5, no. 3. 10.1186/s40900-018-0134-y PMC6330432 30656063

[R33] Reason P & Bradbury H 2001, Handbook of action research: Participative inquiry and practice, Sage Publications, Thousand Oaks.

[R34] Reese AL, Hanza MM, Abbenyi A, Formea C, Meiers SJ, Nigon JA, Osman A, Goodson M, Njeru JW, Boursaw B, Dickson E, Wieland ML, Sia IG & Wallerstein N 2019, ‘The development of a collaborative self-evaluation process for community-based participatory research partnerships using the community-based participatory research conceptual model and other adaptable tools’, Progress in Community Health Partnerships: Research, Education, and Action, vol. 13, no. 3, pp. 225–35. 10.1353/cpr.2019.0050 31564663

[R35] Sandoval JA, Lucero J, Oetzel J, Avila M, Belone L, Mau M, Pearson C, Tafoya G, Duran B, Iglesias Rios L & Wallerstein N 2012, ‘Process and outcome constructs for evaluating community-based participatory research projects: A matrix of existing measures’, Health Education Research, vol. 27, no. 4, pp. 680–90. 10.1093/her/cyr087 21940460 PMC3396879

[R36] Tigges B, Miller D, Dudding KM, Balls-Berry JE, Borawski EA, Gaurav D, Hafer NS, Kimminau KS, Kost RG, Littlefield K, Shannon J & Menon U 2019, ‘Measuring quality and outcome of research collaborations: An integrated review’, Journal of Clinical and Translational Science, vol. 3, no. 5, pp. 261–89. 10.1017/cts.2019.402 31660251 PMC6813516

[R37] United States Census Bureau n.d., QuickFacts, viewed 14 May 2020. https://www.census.gov/quickfacts/

[R38] University of New Mexico (UNM) College of Population Health n.d., CBPR Model, viewed 12 February 2024. https://hsc.unm.edu/population-health/research-centers/center-participatory-research/cbpr-community-engagement/cbprmodel.html https://cpr.unm.edu/research-projects/cbpr-project/cbpr-model.html

[R39] U.S. News & World Reports 2017, ‘Study: Martinsville opioid prescriptions highest in country’, 12 July, https://www.usnews.com/news/best-states/virginia/articles/2017-07-12/study-martinsville-opioid-prescriptions-highest-in-country

[R40] Virginia Department of Health n.d., Opioid Data: Data, viewed 25 June 2025. https://www.vdh.virginia.gov/opioid-data/data/

[R41] Wallerstein NB & Duran B 2006, ‘Using community-based participatory research to address health disparities’, Health Promotion Practice, vol. 7, no. 3, pp. 312–23. 10.1177/1524839906289376 16760238

[R42] Wallerstein N & Duran B 2010, ‘Community-based participatory research contributions to intervention research: The intersection of science and practice to improve health equity’, American Journal of Public Health, vol. 100, suppl. 1, pp. S40–6. 10.2105/AJPH.2009.184036 20147663 PMC2837458

[R43] Wallerstein N, Oetzel J, Duran B, Tafoya G, Belone L & Rae R 2008, ‘What predicts outcomes in CBPR?’, in Minkler M & Wallerstein N (eds), Community Based Participatory Research for Health: Process to Outcomes, 2nd edn, Jossey-Bass, San Francisco, CA, pp. 371–92.

[R44] Wallerstein N, Oetzel JG, Sanchez-Youngman S, Boursaw B, Dickson E, Kastelic S, Koegel P, Lucero JE, Magarati M, Ortiz K, Parker M, Peña J, Richmond A & Duran B 2020, ‘Engage for equity: A long-term study of community-based participatory research and community-engaged research practices and outcomes’, Health Education & Behavior, vol. 47, no. 3, pp. 380–90. 10.1177/1090198119897075 32437293 PMC8093095

[R45] Weiss ES, Anderson RM & Lasker RD, 2002 ‘Making the most of collaboration: exploring the relationship between partnership synergy and partnership functioning’, Health Education & Behavior, vol. 29, no. 6, pp. 683–698. 10.1177/109019802237938 12456129

[R46] Windsor LC 2013, ‘Using concept mapping in community-based participatory research: A mixed methods approach’, Journal of Mixed Methods Research, vol. 7, no. 3, pp. 274–93. 10.1177/1558689813479175 26561484 PMC4638322

[R47] Wold B & Mittelmark MB 2018, ‘Health-promotion research over three decades: The social-ecological model and challenges in implementation of interventions’, Scandinavian Journal of Public Health, vol. 46, suppl. 20, pp. 20–6. 10.1177/1403494817743893 29552963

[R48] Zimmerman EB, Cook SK, Haley AD, Woolf SH & Price SK 2017, ‘A patient and provider research agenda on diabetes and hypertension management’, American Journal Preventative Medicine, vol. 53, no. 1, pp. 123–9. 10.1016/j.amepre.2017.01.034 PMC6979736 28314558

[R49] Zimmerman EB, Cook SK, Woolf SH, Price SK, Haley A, Rafie C & Moser D 2020a, ‘Developing a method for engaging people in setting research agendas’, Patient-Centered Outcomes Research Institute (PCORI). 10.25302/04.2020.ME.131007664 40924851

[R50] Zimmerman EB, Rafie C, Moser DE, Hargrove A, Noe T & Adams Mills C 2020b, ‘Participatory action planning to address the opioid crisis in a rural Virginia community using the SEED Method’, Journal of Participatory Research Methods, vol. 1, no. 1. 10.35844/001c.13182 PMC7451262 32864659

